# Chikungunya Fever Cases Identified in the Veterans Health Administration System, 2014

**DOI:** 10.1371/journal.pntd.0004630

**Published:** 2016-05-04

**Authors:** Tara Perti, Cynthia A. Lucero-Obusan, Patricia L. Schirmer, Mark A. Winters, Mark Holodniy

**Affiliations:** 1 Public Health Surveillance & Research, Department of Veterans Affairs, Washington, D.C., United States of America; 2 Epidemic Intelligence Service, Division of Scientific Education and Professional Development, Centers for Disease Control and Prevention, Atlanta, Georgia, United States of America; 3 Veterans Affairs Palo Alto Health Care System, Palo Alto, California, United States of America; 4 Division of Infectious Diseases and Geographic Medicine, Stanford University, Palo Alto, California, United States of America; Naval Medical Cesearch Center, UNITED STATES

## Abstract

**Background:**

During December 2013, the first locally transmitted chikungunya virus (CHIKV) infections in the Americas were reported in the Caribbean. Although CHIKV infection is rarely fatal, risk for severe disease increases with age and medical comorbidities. Herein we describe characteristics of Veterans Health Administration (VHA) patients with CHIKV infection and, among those with infections diagnosed in Puerto Rico, investigated risk factors for hospitalization.

**Methodology:**

We queried VHA’s national electronic medical records to identify patients with CHIKV testing during 2014. Demographics, clinical history, laboratory results, and outcomes were abstracted. We investigated risk factors for hospitalization among patients with laboratory-confirmed CHIKV infection in Puerto Rico.

**Principal Findings:**

We identified 180 laboratory-confirmed CHIKV infections; 148 (82.2%) were diagnosed in Puerto Rico, and 32 (17.8%) were diagnosed among returning travelers elsewhere in the United States. In Puerto Rico, where more patients were hospitalized (55.4% versus 20.0%) and died (4.1% versus 0%), risk for hospitalization increased with age (relative risk [RR]/each 10-year increase, 1.19; 95% confidence interval [CI], 1.06–1.32) and, adjusted for age, increased among patients with congestive heart failure (RR, 1.58; 95% CI, 1.25–1.99), chronic kidney disease (RR, 1.52; 95% CI, 1.19–1.94), diabetes mellitus (RR, 1.39; 95% CI, 1.06–1.84), or chronic lung disease (RR, 1.37; 95% CI, 1.03–1.82).

**Conclusions/Significance:**

CHIKV infection is an emerging problem among Veterans residing in or visiting areas with CHIKV transmission. Although overall mortality rates are low, clinicians in affected areas should be aware that older patients and patients with comorbidities may be at increased risk for severe disease.

## Author Summary

Infection with mosquito-borne chikungunya virus causes fever and severe diffuse joint pain—an illness known as chikungunya fever, or "that which bends up." Epidemics of chikungunya fever have occurred in Asia, Africa, and Europe. Not until December 2013 were there reports of chikungunya virus infection occurring in the Americas. Since then, it has involved most countries in the Western Hemisphere with >1.1 million cases reported by the end of 2014. Previous data from the Réunion Island outbreak demonstrated that older patients and patients with certain chronic medical conditions may have a higher risk of severe disease. The Veterans Health Administration is the largest health care system in the United States and has facilities in U.S. territories, including Puerto Rico, which has been heavily affected by this epidemic. Among Veterans in Puerto Rico, we investigated risk factors for severe disease and described all chikungunya-associated deaths. Risk for hospitalization increased with age, and for patients of the same age, was increased among those with congestive heart failure, chronic kidney disease, diabetes, or chronic lung disease. Further work is needed to determine whether prevention strategies targeted to those who may be at greatest risk for severe disease could help decrease morbidity and mortality among these populations.

## Introduction

Chikungunya virus (CHIKV), an alphavirus most commonly transmitted by *Aedes* species mosquitoes, causes chikungunya fever (CHIK), characterized by acute-onset of fever and what is frequently described as incapacitating polyarthralgia [1, 2]. Since CHIKV was first identified in Tanzania in 1953 [2], epidemics have occurred in South and Southeast Asia, Africa, and Europe [1, 3, 4]. Three distinct genotypes have been described, including East/Central/South African, Asian, and West African [1, 5]. In December 2013, locally transmitted infection in the Western Hemisphere was first reported; the predominant strain is closely related to the Asian genotype [6–8]. CHIKV has rapidly disseminated among this largely immunologically naïve population; the Pan American Health Organization (PAHO) reported >1.1 million suspect cases, involving the majority of Western Hemisphere countries by the end of 2014 [9]. During 2014, over 31,000 cases (14% laboratory-confirmed) were reported in Puerto Rico [10] and over 1,500 cases (17% laboratory-confirmed) were reported in the U.S. Virgin Islands [11]; these figures are thought to underestimate disease burden because they do not include patients who did not present for care nor those who presented, but were not reported or for whom diagnostic testing was not completed [12]. During 2014, 2,811 laboratory-confirmed infections in the United States were reported to the Centers for Disease Control and Prevention (CDC) through the ArboNET surveillance system; the majority were among returning travelers, except for 12 persons in Florida with locally transmitted infection [13].

CHIK is usually self-limited, with the majority of symptoms typically resolving in 7–10 days [1]; however, patients can have prolonged rheumatologic symptoms [14, 15]. CHIKV infection can also be associated with severe illness, involving neurologic, cardiovascular, respiratory, renal, and ocular manifestations [16]. Although overall mortality is low, estimated at 0.3/1,000 population per year on Réunion Island [17], risk for severe disease and death increases with age and is higher among patients with certain comorbidities [17–19].

The Veterans Health Administration (VHA) has health care facilities throughout the United States and U.S. territories. Because 45% of all U.S. Veterans and 62% of Veterans in Puerto Rico are aged ≥65 years [20], and VHA patients have more comorbidities than Veterans who receive care at non-VHA facilities or non-Veterans [21], VHA patients might be at higher risk for severe CHIK than those in the general U.S. population exposed to the virus (i.e. returning travelers and those living in areas with CHIKV transmission). VHA’s Public Health Surveillance and Research Group (PHSR) performs surveillance for emerging infections among VHA patients. After the CHIK epidemic involved U.S. territories, PHSR began performing CHIK epidemiologic surveillance in July 2014 of all patients with laboratory-confirmed CHIKV infection diagnosed at VHA facilities during 2014. Herein, we describe characteristics of these patients, compare clinical findings with patients who tested negative, investigate risk factors for hospitalization, and report phylogenetic analysis of CHIKV strains detected.

## Methods

We identified patients from all VHA facilities with CHIKV test results for specimens collected during January 1, 2014–December 31, 2014, utilizing VHA’s Corporate Data Warehouse, a national data repository from VHA’s VistA electronic medical record system. Because VHA has no standardized naming convention for laboratory tests and inconsistent application of universal codes that can be utilized for test identification, we queried this data warehouse for any test name containing the term "chik." After distributing CHIKV surveillance reports to VHA facilities beginning in July 2014, PHSR was contacted by VHA facilities that were not listed in the reports but had diagnosed infections. Consequently, we expanded the query to search for "chik" in the comments field to capture additional CHIK diagnostic test results associated with other test names (e.g., “dengue”, “miscellaneous”, and “reference laboratory”). The last query was performed on February 26, 2015.

Apart from VHA’s Public Health Reference Laboratory (PHRL), VHA clinical laboratories do not have CHIK diagnostic testing capability, and thus specimens are sent to non-VHA laboratories for testing. When capacity of non–VHA laboratories in Puerto Rico became overwhelmed by demand [12], not all specimens submitted for CHIKV testing by Veterans Affairs Caribbean Healthcare System (VACHS) in Puerto Rico (1 medical center and 8 outpatient clinics) were processed, including some specimens from patients who subsequently died. To further investigate, we queried the data warehouse for CHIKV testing orders. For patients without results, medical records were reviewed to determine whether tests remained pending, or if cancelled, the reason for cancellation. During December 2014, VHA PHSR provided a list of patients without results to laboratories in Puerto Rico to recover any remaining specimens for testing by PHRL. PHRL performs internally and externally validated CHIKV reverse transcriptase PCR (RT-PCR) (CDC, CHIKV RT-PCR assay protocol, Fort Collins, Colorado) [22] and IgM enzyme-linked immunosorbent assay (ELISA) (Abcam, Inc., Anti-Chikungunya Virus IgM Human ELISA kit, Cambridge, Massachusetts) according to manufacturer’s recommendations. For patients who had specimens collected for CHIKV testing in Puerto Rico or elsewhere in the United States and died before the last query was performed, a physician-epidemiologist reviewed medical records to confirm presenting symptoms were consistent with CHIK (i.e. fever and either oligoarthralgia, polyarthralgia, or myalgia) and the patient resided in or had recently visited an area with CHIKV transmission, and determined whether CHIKV infection might have contributed to death (i.e., the patient had died before recovery from acute illness and death was not clearly from unrelated causes). Although PAHO defines a suspect case as a patient with acute onset of objective fever (>38.5°C or 101.3°F [23, 24]; >38.3°C or 101.0°F [9]; >38.0°C or 100.4°F [9]) and severe arthralgia, not otherwise explained, who resides in or has visited epidemic or endemic areas ≤2 weeks before symptom onset [9, 23, 24], we did not utilize this definition because the majority of our laboratory-confirmed cases did not meet these criteria.

For all patients with available CHIK diagnostic test results, we abstracted demographics, clinical history, including symptoms during the acute illness, laboratory results, and outcomes from medical records. Comorbidities were identified by review of problem lists and provider notes. A laboratory-positive case was defined as a patient with detectable CHIKV RNA by RT-PCR or anti-CHIKV IgM antibody. Patients with positive serology, but without travel to an area with known CHIKV transmission, consistent with a false-positive result, as well as patients with inadequate testing to rule out CHIKV infection (negative RT-PCR >8 days after symptom onset and no serology or negative IgM <4 days after symptom onset and no RT-PCR or convalescent serology) were excluded from statistical analysis [24]. Demographics and clinical characteristics of patients with laboratory-confirmed infection diagnosed in Puerto Rico versus elsewhere in the United States were compared, and clinical findings of CHIKV-positive versus CHIKV-negative patients (all remaining patients with negative CHIKV tests) were compared by using chi-square test for categorical (Fisher’s exact test when n <5 in any cell) and t-test for continuous variables.

To determine whether the association between age and hospitalization was modified by whether CHIK was diagnosed in Puerto Rico versus elsewhere in the United States, analysis was stratified by patient location. Poisson regression with robust error variance [25, 26] was utilized to investigate risk factors for hospitalization among patients with laboratory-confirmed infection in separate age-adjusted models. Potential risk factors investigated included comorbidities, vital signs on presentation, and laboratory abnormalities at presentation and during clinical course. Analyses were performed by using SAS 9.2 (SAS Institute, Inc., Cary, North Carolina).

Finally, we sequenced the CHIKV E1 envelope glycoprotein gene from a convenience sample of patients from Puerto Rico with detectable CHIKV RNA. RNA was extracted from serum by using Qiagen DSP viral RNA mini kits (Qiagen, Germantown, Maryland) according to manufacturer’s instructions. RT-PCR was performed by using Superscript One-Step Platinum Taq HiFi (Thermo Fisher Scientific, Inc., Waltham, Massachusetts) and primers CV1F and CV1R [27]. Resulting PCR products were purified by using Qiagen PCR purification kits and subjected to population-based sequencing by using standard dideoxyterminator techniques. Sequences were assembled, edited, and compared with sequences in GenBank from the Western Hemisphere and previous outbreaks using Geneious software (Biomatters, Inc., San Francisco, California).

### Ethics Statement

This study was reviewed by CDC for human subjects protection and was deemed to be non-research. It was also approved by the Stanford University Institutional Review Board and fulfilled the requirements of regulation OHRP 45 CFR 46.116 (d) for waiver of informed consent. Patient data was anonymized after data abstraction.

## Results

**Fig 1:**
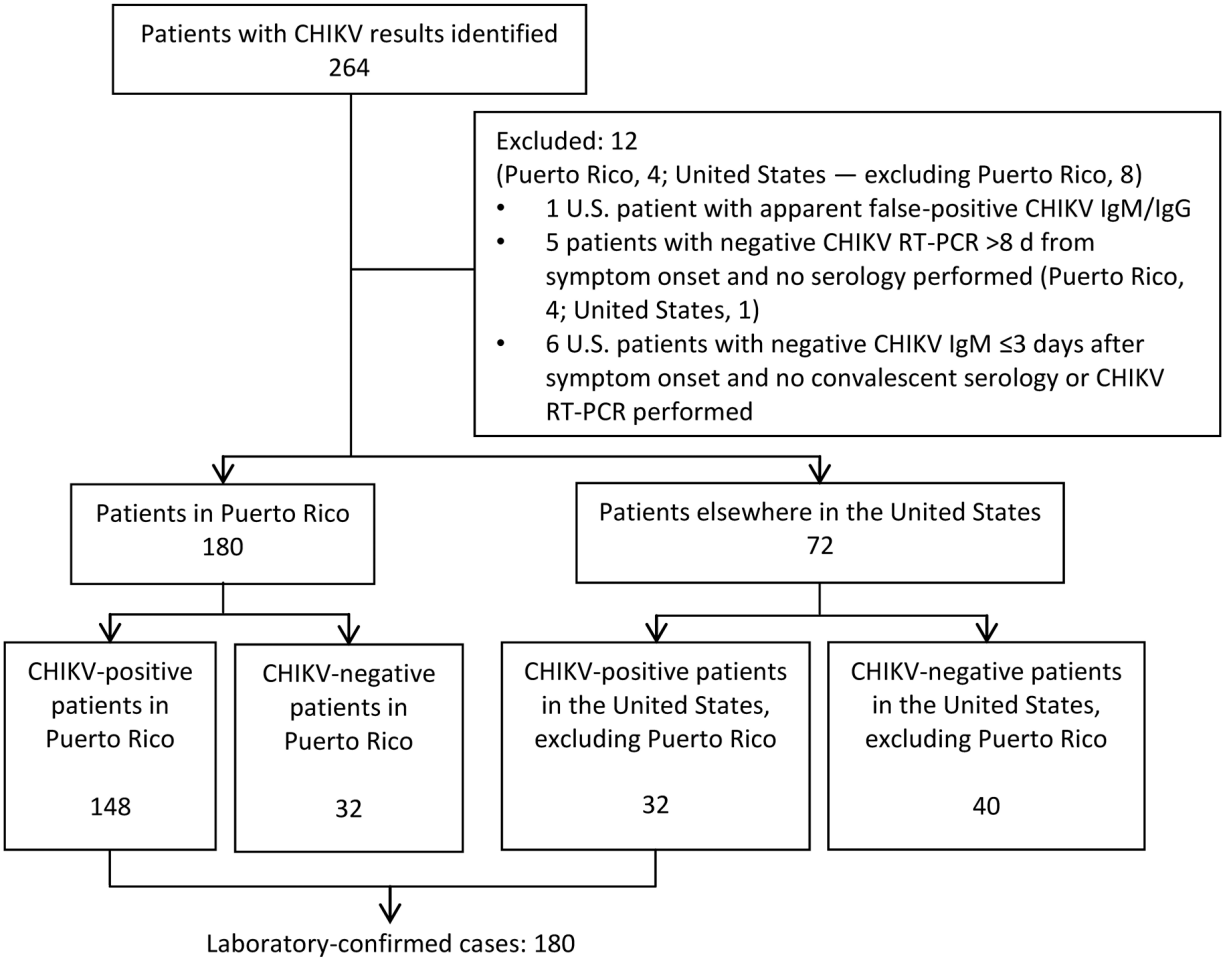
Patients with chikungunya virus test results identified—Veterans Health Administration, 2014. One excluded patient with an apparent false-positive CHIKV IgM/IgG result 4 days after symptom onset had no travel to an area with CHIKV transmission; repeat testing was negative. CHIKV, chikungunya virus; reverse transcriptase PCR (RT-PCR).

**Fig 2:**
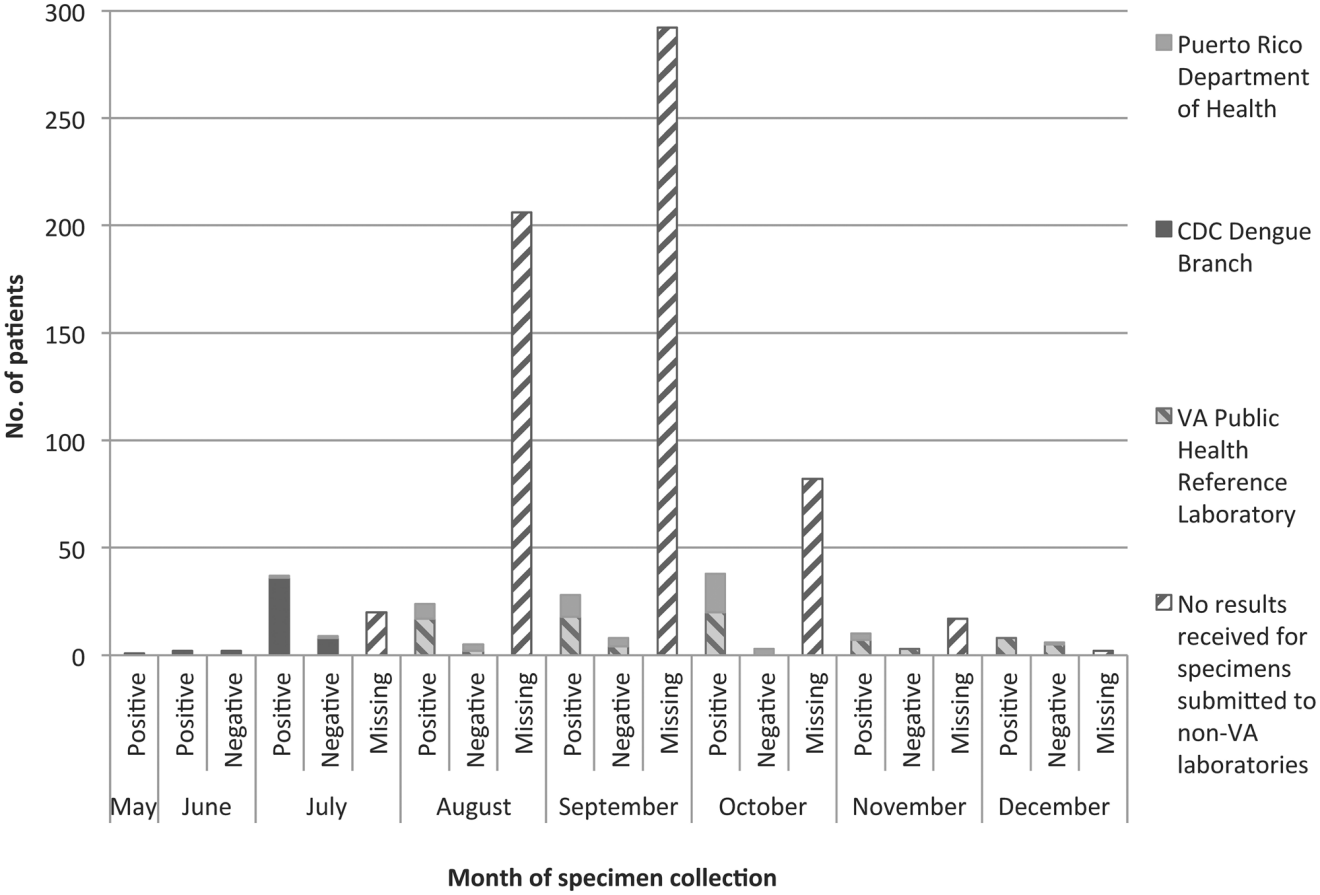
Patients with chikungunya virus test results and patients with specimens submitted for chikungunya virus testing without results—Veterans Affairs Caribbean Healthcare System, 2014 (n = 803). VA PHRL began testing all specimens submitted for chikungunya virus testing from VA Caribbean Healthcare System on December 11, 2014, including specimens for 13 patients prospectively collected during December and unprocessed specimens recovered for 74 patients collected during June–November. Reverse transcriptase PCR results are reported by VA PHRL ≤1 day and serology ≤3 days from specimen receipt.

We identified 264 patients with CHIKV results during 2014 (Fig 1). Results for 184 patients from VACHS in Puerto Rico were available (22.8% of 806 patients from whom specimens were collected for CHIK diagnostic testing; testing was not completed for the remainder) (Fig 2 and S1 Fig). Results for 80 patients were from other U.S. facilities. Twelve patients were excluded; 1 with an apparent false-positive CHIKV IgM/IgG result and 11 with possible false-negative results because of inadequate testing. The remaining 252 patients, including 180 in Puerto Rico and 72 elsewhere in the United States, were included in the analysis.

Overall, 180 patients had laboratory-confirmed CHIKV infection (Table 1). Specimens for CHIKV testing from Puerto Rico were collected a mean of 4.5 d ± 5.4 d from symptom onset. Specimens from the rest of the United States were collected a mean of 28.7 d ± 40.3 d from symptom onset (p = 0.003). In Puerto Rico, 148 (82.2%) of 180 patients tested were CHIKV-positive; 145 (98.0%) were confirmed by RT-PCR and 3 (2.0%) by serology alone. Outside Puerto Rico, 32 (44.4%) of 72 patients tested were CHIKV-positive; 4 (12.5%) were confirmed by RT-PCR and 28 (87.5%) by serology alone. Fourteen (19%) of 72 patients tested for CHIKV infection outside Puerto Rico had no recorded travel to an area with CHIKV transmission prior to symptom onset, all of whom were CHIKV-negative; none of the patients tested in Puerto Rico had symptom-onset prior to possible exposure to circulating CHIKV. Patients with CHIKV infection in Puerto Rico were older (mean, aged 69 versus 55 years; p < .0001) and had more comorbidities than patients outside Puerto Rico.

**Table 1:** Demographics and Clinical Characteristics of Patients with Laboratory-Confirmed Chikungunya Virus Infection—Veterans Health Administration, 2014 (n = 180).

	Puerto Rico n = 148 (82.2%)	United States^a^ n = 32 (17.8%)	P value
Age, mean ± SD, y	68.8 ± 16.2	54.8 ± 16.5	< .0001
Male, no. (%)	142 (96.0)	25 (78.1)	.0004
Race/ethnicity, no. (%)			< .0001
Hispanic	139 (95.2)	9 (32.1)	
White	5 (3.4)	13 (46.4)	
Black	1 (0.7)	6 (21.4)	
Native Hawaiian or Other Pacific Islander	1 (0.7)	0 (0)	
Missing data	2	4	
Comorbidities, no. (%)^b^			
Hypertension	118 (79.7)	13 (40.6)	< .0001
Hyperlipidemia	101 (68.2)	13 (40.6)	.003
Diabetes	62 (41.9)	9 (28.1)	.15
Coronary heart disease	32 (21.6)	3 (9.4)	.14
Congestive heart failure	13 (8.8)	0 (0)	.13
Chronic kidney disease	27 (18.2)	1 (3.1)	.03
COPD or pulmonary fibrosis	16 (10.8)	1 (3.1)	.31
Asthma	16 (10.8)	5 (15.6)	.44
Obstructive sleep apnea	24 (16.2)	4 (12.5)	.79
History of malignancy	23 (15.5)	2 (6.3)	.26
Dementia	19 (12.8)	0 (0)	.03
Immunosuppression	8 (5.4)	0 (0)	.35
CHIKV RT-PCR positivity, no. (%)			< .0001
Positive	145 (98.6)	4 (40.0)	
Negative	2 (1.4)	6 (60.0)	
Not performed	1	22	
CHIKV IgM positivity, no. (%)^c^			< .0001
Positive	23 (32.9)	29 (93.6)	
Negative	47 (67.1)	1 (3.2)	
Equivocal	0 (0)	1 (3.2)	
Not performed	78	1	
Levels of care received, no. (%)^b^			
Primary care provider only	16 (10.8)	10 (33.3)	.001
Emergency department	128 (86.5)	20 (66.7)	.008
Hospitalized	82 (55.4)	6 (20.0)	.0004
Intensive care unit	10 (6.8)	0 (0)	.22
Missing data	0	2	
Died, no. (%)	6 (4.1)	0 (0)	.59
Recent travel among patients who received a diagnosis in the United States (excluding Puerto Rico), no. (%)^d^			
Puerto Rico		11 (34)	
Dominican Republic		8 (25)	
Jamaica		4 (13)	
Haiti		2 (6)	
Dominican Republic and Haiti		2 (6)	
U.S. Virgin Islands		1 (3)	
Trinidad and Tobago		1 (3)	
Philippines		1 (3)	
Guyana		1 (3)	
Ghana		1 (3)	
Reason for travel among patients in the United States, no. (%)			
Visiting family		15 (52)	
Residing in Puerto Rico		7 (24)	
Active-duty deployment		3 (10)	
Civilians working or volunteering		2 (7)	
Vacation		2 (7)	
Missing data		3	

SD, standard deviation; COPD, chronic obstructive pulmonary disease; CHIKV, chikungunya virus; RT-PCR, reverse transcriptase PCR.

^a^ Excluding Puerto Rico.

^b^ Patients can have >1 comorbidity and receive >1 level of care.

^c^ A second serum specimen was obtained for two patients in the United States who were initially seronegative at 1 and 4 days after symptom onset and both specimens demonstrated CHIKV IgM seroconversion, at 37 and 21 days after symptom onset respectively; these results are included under positive CHIKV IgM results. Serologic testing for all but 2 patients in Puerto Rico was performed by the Veterans Health Administration Public Health Reference Lab.

^d^ One patient was diagnosed in Puerto Rico, but had also travelled to the Dominican Republic.

### Clinical and Laboratory Findings

Patients with CHIKV infection frequently reported oligoarthralgia or polyarthralgia (88.3%), subjective fever (84.4%), generalized malaise (76.7%), myalgia (69.4%), and rash (44.4%), and reported these symptoms more often than CHIKV-negative patients (Table 2). Among 165 patients with laboratory-confirmed CHIKV infection who had recorded temperatures during their acute illness, only 53 (32.1%) demonstrated objective fever (>38.0°C or >100.4°F) [9], and only 48 (29.1%) had both objective fever and arthralgia. Subjective fever and arthralgia was reported for 140 (77.8%) of 180 patients and subjective fever or any arthralgia for 174 (96.7%).

**Table 2:** Symptoms and Signs of Patients with Laboratory-Confirmed Chikungunya Virus Infection Compared with Chikungunya Virus-Negative Patients—Veterans Health Administration, 2014.

System	Symptom or Sign	CHIKV-positive (n = 180) no. (%)	CHIKV-negative^a^ (n = 72) no. (%)	P Value
Musculoskeletal^b^	Oligoarthralgia or polyarthralgia	159 (88.3)	45 (62.5)	< .0001
	Myalgia	125 (69.4)	34 (47.2)	.001
	Back pain	26 (14.4)	11 (15.3)	.87
	Neck pain	24 (13.3)	11 (15.3)	.69
	Bone pain	9 (5.0)	0 (0)	.06
Systemic	Fever	152 (84.4)	51 (70.8)	.01
	Generalized malaise or fatigue	138 (76.7)	45 (62.5)	.02
Dermatologic	Rash	80 (44.4)	16 (22.2)	.001
Neurologic	Headache	73 (40.6)	27 (37.5)	.65
	Altered mental status	17 (9.4)	5 (6.9)	.53
	Dizziness	15 (8.3)	3 (4.2)	.29
Gastrointestinal	Anorexia	54 (30.0)	22 (30.6)	.93
	Nausea	48 (26.7)	17 (23.6)	.62
	Vomiting	27 (15.0)	15 (20.8)	.26
	Diarrhea	40 (22.2)	10 (13.9)	.13
	Abdominal pain	24 (13.3)	10 (13.9)	.91
Ophthalmologic	Conjunctival injection	12 (6.7)	1 (1.4)	.12
Pulmonary	Cough	34 (18.9)	21 (29.2)	.07
	Sore throat	16 (8.9)	8 (11.1)	.59
	Dyspnea	14 (7.8)	7 (9.7)	.61
Cardiovascular	Chest pain	21 (11.7)	7 (9.7)	.66
	Edema	13 (7.2)	2 (2.8)	.24
Urologic	Dysuria	9 (5.0)	2 (2.8)	.73
Hematologic	Bruising	3 (1.7)	1 (1.4)	1.0
	Bleeding	7 (3.9)	4 (5.6)	.52

Symptoms and signs during acute illness as recorded in medical records of patients during evaluation for CHIKV infection. Denominator includes all patients, regardless of whether pertinent negative symptoms or signs were recorded. CHIKV, chikungunya virus.

^a^ Alternative diagnoses were recorded for 34 of 72 CHIKV-negative patients as follows: infectious, 17 (influenza, 1; pneumonia, 4; dengue, 2 [41 (56.9%) of CHIKV-negative patients had dengue virus test results]; typhoid fever, 1; Q fever, 1; tickborne infection, 1; cellulitis, 1; deep neck space infection, 1; methicillin-resistant *Staphylococcus aureus* endocarditis, 1; postinfectious arthritis, 2; aseptic meningitis, 1; colitis, 1); rheumatic, 9 (inflammatory arthritis, 4; suspected Sjögren’s syndrome, 1; suspected spondyloarthopathy, 1; mixed connective tissue disease, 1; osteoarthritis and bursitis, 1; myofascial pain, 1); orthopedic, 1 (prosthetic joint loosening); malignant, 2 (lung cancer and viral syndrome, 1; acute myelogenous leukemia, 1); neurologic, 1 (demyelinating polyneuropathy); adverse drug effect, 1; and fever of unknown origin, 3. Over half (53%) of CHIKV-negative patients had no alternative diagnosis recorded or received a diagnosis of viral syndrome of undetermined etiology.

^b^ Of 96 patients with CHIKV infection and recorded arthralgia site (59.3% of 162 with any arthralgia), 59 (61%) reported involvement of the knees, 47 (49%) hands or fingers, 36 (38%) ankles, 32 (33%) shoulders, 30 (31%) wrists, 23 (24%) feet, and 19 (20%) elbows.

Among CHIKV-positive patients, 37.0% of 173 had leukopenia (<4,000 white blood cells [WBC]/μL) and 71.4% of 171 had lymphopenia (<1,000 lymphocytes/μL); these findings occurred more frequently compared with CHIKV-negative patients (Table 3). Thrombocytopenia (<150,000 platelets/μL) occurred among 80 (46.5%) of 172 patients with CHIKV infection, with mean nadir platelet count of 104,000/μL. Acute kidney injury (AKI) (≥0.3 mg/dL or 26.5 μmol/L increase in serum creatinine from last level [28]) was experienced by 33 (21.6%) of 153 patients, 4 (12.1%) of whom had stage III AKI. Hepatic transaminitis (aspartate aminotransferase >40 U/L or alanine aminotransferase >45 U/L) was experienced by 52 (40.3%) of 129 patients, 16 (30.8%) of whom had transaminases >3 times the upper limit of normal.

**Table 3:** Abnormal Laboratory Findings for Patients with Laboratory-Confirmed Chikungunya Virus Infection (n = 180), Compared with Chikungunya Virus-Negative Patients (n = 72)—Veterans Health Administration, 2014.

	CHIKV-positive	CHIKV-Negative	P Value
Leukopenia (n = 239), no. (%)	**64 (37.0)**	**15 (22.7)**	**.04**
WBC on presentation (n = 78), mean ± SD, cells ×10^3^/μL	4.6 ± 1.4	5.3 ± 3.8	
WBC nadir (n = 79), mean ± SD, cells ×10^3^/μL	2.9 ± 0.6	3.0 ± 0.7	
Lymphopenia (n = 234), no. (%)	**122 (71.4)**	**24 (38.1)**	**< .0001**
ALC on presentation (n = 145), mean ± SD, cells ×10^3^/μL	0.6 ± 0.3	0.7 ± 0.2	
ALC nadir (n = 146), mean ± SD cells ×10^3^/μL	0.6 ± 0.2	0.6 ± 0.2	
Thrombocytopenia (n = 239), no. (%)	80 (46.5)	28 (41.8)	.51
Platelet count on presentation (n = 106), mean ± SD, ×10^3^cells/μL	121 ± 35	121 ± 39	
Platelet count nadir (n = 108), mean ± SD, ×10^3^cells/μL	104 ± 31	93 ± 39	
Acute kidney injury^a^ (n = 213), no. (%)	33 (21.6)	9 (15.0)	.28
Stage I	23 (69.7)	8 (88.9)	
Stage II	6 (18.2)	0 (0)	
Stage III	4 (12.1)	1 (11.1)	
Hepatic transaminitis (n = 184), no. (%)	52 (40.3)	23 (41.8)	.85
1–2 × ULN	29 (55.8)	10 (43.5)	
2–3 × ULN	7 (13.5)	8 (34.8)	
>3 × ULN	16 (30.8)	5 (21.7)	

CHIKV, chikungunya virus; WBC, white blood cell count; SD, standard deviation; ALC, absolute lymphocyte count; and ULN, upper limit of normal.

^a^Acute kidney injury stage I, increase in serum creatinine by ≥0.3 mg/dL or 26.5 μmol/L from baseline (last creatinine); stage II, 2.0–2.9 × baseline; and stage III, ≥3.0 × baseline [28].

### Risk Factors Associated with Hospitalization

In Puerto Rico, 82 (55.4%) of 148 patients with laboratory-confirmed CHIKV infection were hospitalized, including 10 (6.8%) who required intensive care; elsewhere in the United States, 6 (20.0%) of 30 returning travelers with known hospitalization status were hospitalized (p = .0004). Whereas the hospitalization rate increased with age among patients in Puerto Rico (relative risk [RR]/ each 10-year increase in age, 1.19; 95% confidence interval [CI], 1.06–1.32), it did not increase with age among returning travelers. Because only 6 returning travelers were hospitalized, analysis is only presented for patients in Puerto Rico (Table 4). After adjusting for age, a significantly higher risk for hospitalization associated with having congestive heart failure (CHF) (RR, 1.58; 95% CI, 1.25–1.99), chronic kidney disease (CKD) (RR, 1.52; 95% CI, 1.19–1.94), diabetes mellitus (RR, 1.39; 95% CI, 1.06–1.84), or chronic lung disease (RR, 1.37; 95% CI, 1.03–1.82) remained. Adjusted for age, patients had a greater risk for hospitalization if they were tachycardic (>100 beats/minute; RR, 1.49; 95% CI, 1.12–1.98), had leukocytosis (>11,000 WBC/μL; RR, 1.65; 95% CI, 1.34–2.03), AKI (RR, 1.64; 95% CI, 1.33–2.04), or hepatic transaminitis (RR, 1.38; 95% CI, 1.07–1.80) at presentation.

**Table 4:** Risk Factors Associated with Hospitalization Among Patients with Laboratory-Confirmed Chikungunya Virus Infection—Veterans Affairs Caribbean Healthcare System, 2014.

	Hospitalization			
	Unadjusted RR^a^	P Value	RR, adjusted for age^a^	P Value
Age, per 10-year increase	**1.19 (1.06–1.32)**	**.002**		
**Comorbidities**				
Congestive heart failure	1.78 (1.42–2.23)	< .0001	**1.58 (1.25–1.99)**	**.0001**
Coronary heart disease	1.41 (1.07–1.87)	0.02	1.18 (0.88–1.58)	.28
Chronic kidney disease	1.77 (1.40–2.25)	< .0001	**1.52 (1.19–1.94)**	**.0008**
Diabetes mellitus	1.53 (1.15–2.03)	.004	**1.39 (1.06–1.84)**	**.02**
Hypertension	1.35 (0.87–2.09)	.18	1.00 (0.63–1.57)	.99
Chronic obstructive pulmonary disease or pulmonary fibrosis	1.55 (1.17–2.07)	.003	**1.37 (1.03–1.82)**	**.03**
Asthma	1.15 (0.76–1.73)	.52	1.40 (0.92–2.14)	.12
Obstructive sleep apnea	1.16 (0.82–1.64)	.41	1.40 (1.00–1.95)	.05
Dementia	1.52 (1.14–2.02)	.004	1.21 (0.90–1.63)	.21
History of malignancy	1.32 (0.96–1.81)	.09	1.17 (0.85–1.61)	.35
Immunosuppression	0.90 (0.44–1.82)	.76	0.97 (0.53–1.77)	.93
**Findings at presentation**				
Tachycardia	1.35 (1.00–1.82)	.049	**1.49 (1.12–1.98)**	**.006**
Fever (>101.0°F or >38.3°C)	0.93 (0.61–1.42)	.74	0.96 (0.62–1.49)	.85
Leukocytosis	1.91 (1.63–2.25)	< .0001	**1.65 (1.34–2.03)**	**< .0001**
Leukopenia	1.12 (0.77–1.61)	.56	1.14 (0.79–1.65)	.49
Lymphopenia	0.83 (0.61–1.12)	.22	0.80 (0.60–1.08)	.14
Thrombocytopenia	1.39 (1.05–1.85)	.02	1.27 (0.96–1.68)	.09
Acute kidney injury	1.80 (1.48–2.19)	< .0001	**1.64 (1.33–2.04)**	**< .0001**
Hepatic transaminitis	1.44 (1.10–1.87)	.007	**1.38 (1.07–1.80)**	**.01**
**Findings during clinical course**				
Thrombocytopenia	1.66 (1.22–2.27)	.001	**1.55 (1.15–2.10)**	**.004**
Acute kidney injury	1.88 (1.52–2.32)	< .0001	**1.75 (1.40–2.19)**	**< .0001**
Hepatic transaminitis	1.50 (1.15–1.97)	.003	**1.44 (1.10–1.88)**	**.008**

Missing data, findings on presentation: tachycardia (n = 2), fever (n = 2), leukocytosis (n = 4), leukopenia (n = 4), lymphopenia (n = 4), thrombocytopenia (n = 5), acute kidney injury (n = 14), hepatic transaminitis (n = 39); findings during clinical course: thrombocytopenia (n = 3), acute kidney injury (n = 13), and hepatic transaminitis (n = 36). RR, relative risk.

^a^ Reference groups for all categorical variables were patients with laboratory-confirmed CHIKV infection without the condition indicated.

### Atypical Features

Two patients with CHIKV infection, confirmed by RT-PCR, developed septic shock without another identified etiology. One patient, with CHIKV infection confirmed by RT-PCR of serum, developed meningitis with a CSF profile consistent with a viral etiology, demonstrating pleocytosis (30 WBC/mm^3^) with initial predominant monocytosis and elevated protein (88 mg/dL). CSF was not submitted for CHIKV testing and no CSF was recovered for diagnostic testing by VA PHRL. One patient presented with Guillain-Barré syndrome one month after CHIK; recent CHIKV infection was confirmed by positive IgM serology. Two patients with CHIKV infection, confirmed by RT-PCR, presented with diabetic ketoacidosis; one patient with CHIKV infection, confirmed by RT-PCR, presented with pancreatitis; one patient with CHIKV infection, confirmed by RT-PCR, presented with colitis, and one patient with CHIKV infection, confirmed by RT-PCR of serum, experienced monomicrobial nonneutrocytic ascites (recovered peritoneal fluid collected 20 days after symptom onset was CHIKV IgM and RT-PCR negative). Seven patients with CHIKV infection (6 confirmed by RT-PCR and 1 by IgM) experienced pneumonia during their clinical course. Four patients with CHIKV infection, confirmed by RT-PCR, experienced congestive heart failure exacerbations; three patients with CHIKV infection, confirmed by RT-PCR, presented with syncope; and one patient with CHIKV infection, confirmed by RT-PCR, experienced a non ST-elevation myocardial infarction. One patient with CHIKV infection, confirmed by RT-PCR, presented with epididymitis.

### Fatalities Associated with Confirmed and Suspected CHIKV Infections in Puerto Rico

Among the 148 patients with laboratory-confirmed CHIKV infection, 6 (4.1%) died. All had viremia demonstrated by RT-PCR (Table 5). In addition to these six patients, there were 15 patients who died after presenting with an illness compatible with CHIK that may have contributed to death; specimens for these patients were submitted for CHIKV testing but were not processed. Although attempts were made to obtain these specimens submitted to non-VHA laboratories, they were not recovered for testing. These 15 patients are thus considered suspect cases (data available upon request).

**Table 5:** Laboratory-Confirmed Chikungunya-Associated Fatal Cases—Veterans Affairs Caribbean Healthcare System, 2014.

	Presenting Symptoms and Signs									
Age (yrs)	Days from Symptom Onset	Symptoms and Signs	Temp (°F)	Heart Rate (beats /min)	Medical Comorbidities	Atypical Manifestations of CHIKV Infection	Additional Diagnostic Laboratory Testing	Additional Complications	Duration of Hospitalization (days)	Cause of Death
82	5	Generalized malaise, weakness, polyarthralgia, arthritis, myalgia, AMS, myoclonus, petechial rash	98.2	123	DM, atrial fibrillation, HTN, HLD, prostate cancer	Meningoencephalitis; atrial fibrillation with RVR; NSTEMI; stage III AKI; hepatic transaminitis [on presentation AST, 93 U/L (peak 146 U/L) and ALT, 29 U/L (peak 93 U/L)]; thrombocytopenia (PLT, 97,000/μL on presentation); septic shock	CSF 6 days after symptom onset: 30 WBC/mm^3^, 61% monocytes, 28% lymphocytes, 11% PMNs; 30 RBC/mm^3^; protein, 88 mg/dL; glucose, 70 mg/dL; opening pressure, 26 cm H_2_O; 9 days after symptom onset: 10 WBC/mm^3^, 70% lymphocytes, 30% PMNs; 121 RBC/mm^3^; protein, 63 mg/dL; glucose, 56 mg/dL. CSF HSV PCR negative. CSF VDRL NR. CSF, blood, and urine cultures negative. CSF AFB and fungal cultures negative. CSF cryptococcal antigen negative. HIV negative. WNV, EBV, and Toxoplasma serology demonstrated past infection. Leptospirosis serology negative. DENV IgM serology negative.	Respiratory failure	23	Meningo-encephalitis
83	1	Polyarthritis, myalgia, AMS, ecchymosis	99.1	123	Alcohol dependence	Thrombocytopenia (PLT nadir 4 d after symptom onset, 92,000/μL, decreased from 152,000/μL on presentation); hepatic transaminitis [on presentation AST, 87 U/L (peak 128 U/L) and ALT, 21 U/L (peak 36 U/L)]	CSF 13 days after symptom onset and 12 days after hospital admission: 237 WBC/mm^3^, 90% PMNs; 30,000 RBC/mm^3^; protein, 218 mg/dL; glucose, 63 mg/dL; bacterial CSF cultures, after broad-spectrum antibiotics for HAP, were negative. CSF AFB and fungal cultures negative. CSF VDRL NR. CSF HSV PCR negative. Blood cultures negative, HIV negative. DENV PCR and IgM serology negative.	Alcohol withdrawal; meningo-encephalitis (treated empirically for bacterial meningitis); HAP; stroke; candidemia	99 (including hospice care at CLC)	Possible sepsis (patient in hospice care at time of death; living independently prior to admission for CHIK)
68	5	Fever, generalized malaise, weakness, oligoarthralgia, myalgia, back pain, headache, photophobia, anorexia, N/V, melena, maculopapular rash	97.8	102	DM, HTN, HLD	Septic shock with multiorgan failure (stage III AKI, ischemic hepatitis (AST 1373 U/L, ALT 400 U/L), NSTEMI, severe acidemia secondary to lactic acidosis, suspected DIC (platelet count 64,000/μL, INR 1.39, PTT 34.6 sec), WBC 56,000/μL	Blood and urine cultures negative; DENV IgM serology negative.		1	Septic shock
70	1	Fever, myalgia, AMS, ecchymosis, hematuria	99.4	73	Cirrhosis due to hemo-chromatosis and alcohol dependence, DM, CKD, HTN, dementia, chronic foley catheter	AKI (Stage II on presentation, progressed to Stage III); thrombocytopenia (PLT nadir 3 days after symptom onset, 56,000/μL, decreased from 81,000/μL on presentation); hepatic transaminitis (on presentation AST, 64 U/L and ALT, 43 U/L)	Blood culture negative; urine culture: *Klebsiella pneumoniae*; DENV IgM serology positive and DENV PCR negative (4 days after symptom onset).	UTI on presentation; DKA; hepatic encephalo-pathy; HAP	29 (including hospice care at CLC)	Acute on chronic kidney injury; hepatic encephalo-pathy
99	7	Fever, weakness, pharyngitis, constipation	99.3	129	CHD, HTN, HLD, dementia	Stage I AKI; thrombocytopenia (PLT nadir 8 days after symptom onset, 120,000/μL); hepatic transaminitis (AST 127 U/L and ALT 47 U/L on presentation)	Blood cultures negative; DENV PCR and IgM negative.	Readmitted for HCAP 3 days after discharge for CHIK	14 (from time of first admission)	Possible aspiration pneumonia
66	4	Fever, generalized malaise, arthralgia, myalgia, AMS, headache, chest and abdominal pain, jaundice, anorexia, N/V, diarrhea, hematemesis; household contact with CHIK	97.8	79	Cirrhosis due to HCV with baseline thrombo-cytopenia, DM, HTN, carcinoid tumor	Decompensated chronic liver disease	Blood culture negative; urine culture: *Escherichia coli*; DENV PCR and IgM negative.	UTI; stage III AKI; femoral fracture after fall.	12	Liver failure (suspected precipitation by acetamino-phen use for CHIKV-related arthralgia and myalgia)

All confirmed cases had chikungunya virus viremia demonstrated by reverse transcriptase PCR. AMS, altered mental status; DM, diabetes mellitus; HTN, hypertension; HLD, hyperlipidemia; RVR, rapid ventricular response; NSTEMI, non-ST elevation myocardial infarction; AKI, acute kidney injury; AST, aspartate aminotransferase; ALT, alanine aminotransferase; PLT, platelet count; CSF, cerebrospinal fluid; WBC, white blood cell; PMNs, polymorphonuclear leukocytes; RBC, red blood cell; HSV, herpes simplex virus; VDRL, Venereal Disease Research Laboratory test; NR; non-reactive; AFB, acid-fast bacilli; WNV, West Nile virus; EBV, Epstein-Barr virus; DENV, dengue virus; HAP, hospital-acquired pneumonia; CLC, Community Living Center (VA nursing home); CHIK, chikungunya fever; N/V, nausea and vomiting; DIC, disseminated intravascular coagulation; INR, international normalized ratio; PTT, partial thromboplastin time; CKD, chronic kidney disease; UTI, urinary tract infection; DKA, diabetic ketoacidosis; CHD, coronary heart disease; HCAP, healthcare-associated pneumonia; HCV, Hepatitis C virus; CHIKV, chikungunya virus.

The mean age of patients with laboratory-confirmed CHIKV infection in Puerto Rico who died was 78 years (range, 66–99 years), compared with 68 years (range, 23–94 years) for those in Puerto Rico who survived (p = 0.12). All 6 patients who died were afebrile on presentation (only one had a recorded temperature >100.4°F or >38.0°C during hospitalization); four were tachycardic on presentation. Of 5 with known medical history, all had multiple comorbidities.

### Phylogenetic Analysis

**Fig 3:**
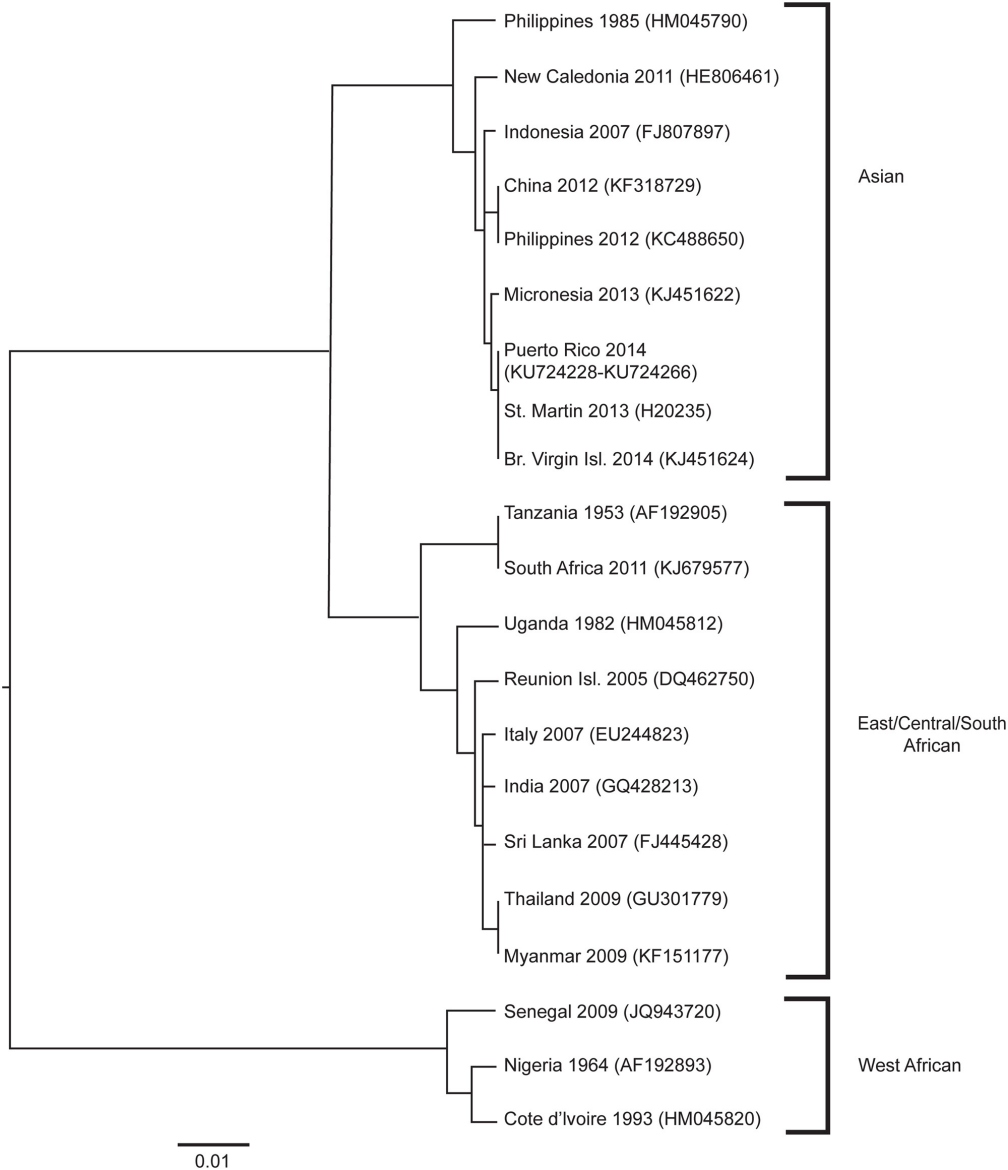
Phylogenetic tree comparing sequences of chikungunya virus strains from Puerto Rico with strains from other regions. Sequences (700 bp) from the E1 envelope glycoprotein gene were aligned and compared in a neighbor-joining tree with a Jukes-Cantor model and 1000 bootstrapping replicates. The resulting tree, with GenBank accession numbers, was displayed by using FigTree v1.2 (http://tree.bio.ed.ac.uk/software/figtree/). The scale bar indicates the distance corresponding with 0.01 nucleotide substitutions per site.

CHIKV E1 envelope glycoprotein gene sequences (GenBank accession numbers: KU724228-KU724266) for 39 patients in Puerto Rico were closely related (<0.5% nucleotide difference) and nearly identical to strains in GenBank from St. Martin and the British Virgin Islands (Fig 3) [6, 7].

## Discussion

Our study is the first to characterize U.S. Veterans with laboratory-confirmed CHIKV infection. The majority received a diagnosis in Puerto Rico, although 32 were returning travelers who received a diagnosis elsewhere in the United States. Although the majority reported subjective fever, only one-third had objective fever, indicating that use of recommended CHIK case definitions, requiring objective fever, might underestimate disease burden among VHA patients [24]. Among returning travelers, approximately one-fifth were hospitalized, whereas in Puerto Rico, where patients were older and had more comorbidities, approximately half of laboratory-confirmed patients were hospitalized, 6.8% required intensive care, and 4.1% died. The fatality rate was higher among VHA patients compared with a preliminary report from Puerto Rico in December 2014 that described only 4 deaths identified by passive surveillance on the island [12]. Although we performed population sequencing of only a portion of the E1 envelope glycoprotein gene, we did not find substantial difference among sequences from Puerto Rico compared with other strains circulating in the Western Hemisphere [6, 7]. As in previous studies [18, 19], we report that age was associated with increased risk for hospitalization. After adjusting for age, CHF (but not coronary heart disease), CKD, diabetes, and chronic lung disease were associated with increased risk for hospitalization. While we cannot be certain of the individual physicians' criteria for hospitalization in many cases, we do know that patients were more likely to be hospitalized if they had unstable vital signs (e.g. tachycardia) or had abnormal laboratory results (e.g. leukocytosis, acute kidney injury, or hepatic transaminitis) at presentation.

Although literature review demonstrates low overall mortality, among persons with CHIKV infection presenting for care, the case fatality rate is not insignificant. During the CHIKV outbreak on Réunion Island (East/Central/South African genotype) [29], among 157 patients with laboratory-confirmed infection who presented to a medical center, 61.8% were hospitalized and 3.2% died [18]. Although patients in that study were >10 years younger and fewer had comorbidities, hospitalization and case fatality rates were similar to VACHS. They reported that diabetes and ischemic heart disease, unadjusted for age, was associated with increased odds of hospitalization [18]; only 11 patients in that study had CKD. Economopoulou et al. reported that among a subset of hospitalized patients with CHIK, cardiac disease, respiratory disease, as well as hypertension were associated with increased risk for severe disease [19].

Because our study included patients with laboratory-confirmed CHIKV infection, we cannot assess the overall burden of CHIKV infection among Veterans, only those who presented to VHA facilities and had appropriate diagnostic testing completed. During 2014, investigation of households surrounding laboratory-positive cases demonstrated that 28% of participants were laboratory-positive for current or recent CHIKV infection, and only 63% of symptomatic persons had sought care [12]. Although our query was robust, any patient with results not entered into the laboratory component of the electronic medical record (e.g., scanned or recorded in a progress note) would not have been captured. Sample size limited analysis of returning travelers, and in Puerto Rico, prevented inclusion of >1 comorbidity in age-adjusted models or assessment of risk factors for intensive care or death among patients with laboratory-confirmed infection. This study identified the lack of testing availability for VACHS, as well as deficiencies in CHIKV diagnostic testing across VHA. We identified 11 patients (4 in Puerto Rico and 7 elsewhere in the United States) with inadequate testing to diagnose CHIKV infection, which may have contributed to underdiagnosis of CHIKV-infection. In Puerto Rico this was because of underuse of serology for patients who presented >8 days after symptom onset. Outside Puerto Rico this was because of underuse of CHIKV RT-PCR or convalescent serology for patients who presented during the first week of symptom onset. Among returning travelers, many of whom presented for care in the U.S. during the convalescent period, when diagnosis is dependent upon serology, some diagnoses could have been missed as CHIKV IgM typically declines after several weeks to months [1]. Only 4 of the returning travelers had CHIKV IgM performed without simultaneous CHIKV IgG, and no patients had a negative CHIKV IgM and positive CHIKV IgG, however, suggesting that few cases may have been missed for this reason. Outside Puerto Rico, only 44% of patients tested for CHIKV had laboratory-confirmed infection; this not only reflects the lower prevalence of CHIKV infection outside Puerto Rico, but also improper testing of patients without symptom-onset after travel to an area with CHIKV transmission. Our surveillance activities determined that CHIKV testing availability for VACHS was lacking and resulted in VHA PHRL offering CHIKV (and dengue virus) testing. Further education of VHA providers regarding CHIKV infection, correct diagnostic testing (RT-PCR versus serology) on the basis of time from symptom onset, and available testing through VHA is needed.

Lessons learned from Puerto Rico are important for areas in the Western Hemisphere with ongoing CHIKV transmission as well as countries, including the United States, with similarly immunologically naïve populations and *Aedes aegypti* or *Aedes albopictus* vectors [6]. For clinical management, newly required CHIK public health reporting, and surveillance, having adequate laboratory testing capacity for timely results is helpful. Although testing all symptomatic persons might be infeasible, sufficient capacity to test those with severe (e.g. hospitalized patients) or atypical illness is needed. Clinicians practicing in areas with CHIKV transmission should be aware that CHIKV infection among elderly patients and patients with comorbidities, including CHF, CKD, diabetes, and chronic lung disease may be associated with more severe disease. To determine whether the risk of atypical complications is greater for CHIKV infection compared with other viral infections, a larger cohort of patients presenting with a viral syndrome would need to be studied. Further work to examine risk factors for intensive care and death among a larger sample of patients with laboratory-confirmed infection is needed to provide closer monitoring for those at greatest risk and to investigate the effect of prevention strategies [30] targeted to populations at greatest risk should they acquire CHIKV infection.

## Supporting Information

S1 Fig Results received for patients who had specimens collected for chikungunya virus testing—Veterans Affairs Caribbean Healthcare System, 2014. Of 806 patients who had specimens collected for chikungunya virus testing, 184 (22.8%) were tested. Of 716 patients who had specimens submitted to non-Veterans Health Administration laboratories in Puerto Rico for chikungunya virus testing, results were received for 97 (13.5%). Reasons provided for rejected specimens (n = 32) included incomplete information (symptom onset date or date of birth not specified), 29; no case investigation form, 1; discrepancy between specimen and form, 1; and unknown, 1. CHIKV, chikungunya virus; VHA, Veterans Health Administration; PHRL Public Health Reference Laboratory; and PR DoH, Puerto Rico Department of Health.
(TIF)

## References

[1] Pialoux G, Gauzere BA, Jaureguiberry S, Strobel M. Chikungunya, an epidemic arbovirosis. Lancet Infect Dis. 2007;7: 319–27. 17448935 10.1016/S1473-3099(07)70107-X

[2] Robinson MC. An epidemic of virus disease in Southern Province, Tanganyika Territory, in 1952–53. I. Clinical features. Trans R Soc Tropical Med Hyg. 1955;49: 28–32. 10.1016/0035-9203(55)90080-8 14373834

[3] Thiberville SD, Moyen N, Dupuis-Maguiraga L, Nougairede A, Gould EA, Roques P, et al Chikungunya fever: epidemiology, clinical syndrome, pathogenesis and therapy. Antiviral Res. 2013;99: 345–70. 10.1016/j.antiviral.2013.06.009 23811281 PMC7114207

[4] Delisle E, Rousseau C, Broche B, Leparc-Goffart I, G LA, Cochet A, et al Chikungunya outbreak in Montpellier, France, September to October 2014. Euro Surveill. 2015;20:17. 25955774 10.2807/1560-7917.es2015.20.17.21108

[5] Powers AM, Brault AC, Tesh RB, Weaver SC. Re-emergence of Chikungunya and O'nyong-nyong viruses: evidence for distinct geographical lineages and distant evolutionary relationships. J Gen Virol. 2000;81: 471–9. 10644846 10.1099/0022-1317-81-2-471

[6] Leparc-Goffart I, Nougairede A, Cassadou S, Prat C, de Lamballerie X. Chikungunya in the Americas. Lancet. 2014;383: 514 10.1016/S0140-6736(14)60185-9 24506907

[7] Lanciotti RS, Valadere AM. Transcontinental movement of Asian genotype chikungunya virus. Emerg Infect Dis. 2014;20: 1400–2. 10.3201/eid2008.140268 25076384 PMC4111183

[8] Teixeira MG, Andrade AM, Costa Mda C, Castro JN, Oliveira FL, Goes CS, et al East/Central/South african genotype chikungunya virus, Brazil, 2014. Emerg Infect Dis. 2015;21: 906–7. 10.3201/eid2105.141727 25898939 PMC4412231

[9] Pan American Health Organization. Number of reported cases of chikungunya fever in the Americas, by country or territory 2013–2014—cumulative cases. 2015 May 15 [cited 2015 May 22]. Available: http://www.paho.org/hq/index.php?option=com_topics&view=readall&cid=5927&Itemid=40931&lang=en.

[10] Puerto Rico Department of Health. Informe semanal de vigilancia de chikungunya: reporte ChikV semana 18–2015. 2015 May 20 [cited 2015 May 22]. Available: http://www.salud.gov.pr/Estadisticas-Registros-y-Publicaciones/Estadisticas%20Chikungunya/Reporte%20ChikV%20Semana%2018-2015.pdf.

[11] US Virgin Islands Department of Health. USVI chikungunya surveillance weekly report- week 53. 2015 January 22 [cited 2015 May 22]. Available: http://www.healthvi.org/news/chik-weekly-report/wk53_chikungunyasurveillanceweeklyreport_usvi.pdf.

[12] Sharp TM, Roth NM, Torres J, Ryff KR, Perez Rodriguez NM, Mercado C, et al Chikungunya cases identified through passive surveillance and household investigations—Puerto Rico, May 5–August 12, 2014. MMWR Morb Mortal Wkly Rep. 2014;63: 1121–8. 25474032 PMC4584601

[13] Centers for Disease Control and Prevention. Chikungunya virus: 2014 final data for the United States. 2015 [cited 2015 Oct 30]. Available: http://www.cdc.gov/chikungunya/geo/united-states-2014.html

[14] Sissoko D, Malvy D, Ezzedine K, Renault P, Moscetti F, Ledrans M, et al Post-epidemic Chikungunya disease on Réunion Island: course of rheumatic manifestations and associated factors over a 15-month period. PLoS Negl Trop Dis. 2009;3:e389 10.1371/journal.pntd.0000389 19274071 PMC2647734

[15] Brighton SW, Prozesky OW, de la Harpe AL. Chikungunya virus infection. A retrospective study of 107 cases. S Afr Medical Journal. 1983;63: 313–5. 6298956

[16] Rajapakse S, Rodrigo C, Rajapakse A. Atypical manifestations of chikungunya infection. Trans R Soc Trop Med Hyg. 2010;104: 89–96. 10.1016/j.trstmh.2009.07.031 19716149

[17] Renault P, Solet JL, Sissoko D, Balleydier E, Larrieu S, Filleul L, et al A major epidemic of chikungunya virus infection on Réunion Island, France, 2005–2006. Am J Trop Med Hyg. 2007;77: 727–31. 17978079

[18] Borgherini G, Poubeau P, Staikowsky F, Lory M, Le Moullec N, Becquart JP, et al Outbreak of chikungunya on Réunion Island: early clinical and laboratory features in 157 adult patients. Clin Infect Dis 2007;44: 1401–7. 17479933 10.1086/517537

[19] Economopoulou A, Dominguez M, Helynck B, Sissoko D, Wichmann O, Quenel P, et al Atypical Chikungunya virus infections: clinical manifestations, mortality and risk factors for severe disease during the 2005–2006 outbreak on Réunion. Epidemiol Infect 2009;137: 534–41. 10.1017/S0950268808001167 18694529

[20] U.S. Department of Veterans Affairs. The Veteran population projection model 2014 (VetPop2014). 2015 [cited 2015 February 25]. Available: http://www.va.gov/vetdata/Veteran_Population.asp

[21] Agha Z, Lofgren RP, VanRuiswyk JV, Layde PM. Are patients at Veterans Affairs medical centers sicker? A comparative analysis of health status and medical resource use. Arch Intern Med. 2000;160: 3252–7. 11088086 10.1001/archinte.160.21.3252

[22] Lanciotti RS, Kosoy OL, Laven JJ, Panella AJ, Velez JO, Lambert AJ, et al Chikungunya virus in US travelers returning from India, 2006. Emerg Infect Dis. 2007;13: 764–7. 17553261 10.3201/eid1305.070015 PMC2738459

[23] Pan American Health Organization. Information for healthcare providers: chikungunya fever; 2014. 2014 [cited 2015 February 24]. Available: http://www.paho.org/hq/index.php?option=com_topics&view=readall&cid=5511&Itemid=40931&lang=en.

[24] Pan American Health Organization. Preparedness and response for chikungunya virus: introduction in the Americas. 2011. [cited 2014 November 3]. Available: http://www.paho.org/HQ/index.php?option=com_content&view=article&id=3545:preparedness-response-chikungunya-virus-introduction-americas&Itemid=40377&lang=en.

[25] Zou G. A modified poisson regression approach to prospective studies with binary data. Amer J Epidemiol. 2004;159: 702–6. 15033648 10.1093/aje/kwh090

[26] Lindquist K. How can I estimate relative risk in SAS using proc genmod for common outcomes in cohort studies? Los Angeles: Institute for Digitial Research and Education, University of California; updated 2015 [cited 2015 Feb 18]. Available: http://www.ats.ucla.edu/stat/sas/faq/relative_risk.htm.

[27] Thiberville SD, Boisson V, Gaudart J, Simon F, Flahault A, de Lamballerie X. Chikungunya fever: a clinical and virological investigation of outpatients on Réunion Island, South-West Indian Ocean. PLoS Negl Trop Dis. 2013;7:e2004 10.1371/journal.pntd.0002004 23350006 PMC3547841

[28] Kidney Disease: Improving Global Outcomes (KDIGO). Acute Kidney Injury Work Group. KDIGO clinical practice guideline for acute kidney injury. Kidney Inter. (2):Suppl 2012 [cited 2015 Feb 18]. Available: http://kdigo.org/home/guidelines/acute-kidney-injury/.

[29] Morrison TE. Reemergence of chikungunya virus. J Virol. 2014;88:11644–7. 10.1128/JVI.01432-14 25078691 PMC4178719

[30] Weaver SC, Lecuit M. Chikungunya virus and the global spread of a mosquito-borne disease. N Engl J Med. 2015;372:1231–9. 10.1056/NEJMra1406035 25806915

