# Supplementary figures and images for: Chikungunya Fever Cases Identified in the Veterans Health Administration System, 2014
Source: PLoS Negl Trop Dis. 2016 May 4;10(5):e0004630. doi: 10.1371/journal.pntd.0004630 (PMC4856344; doi:10.1371/journal.pntd.0004630)

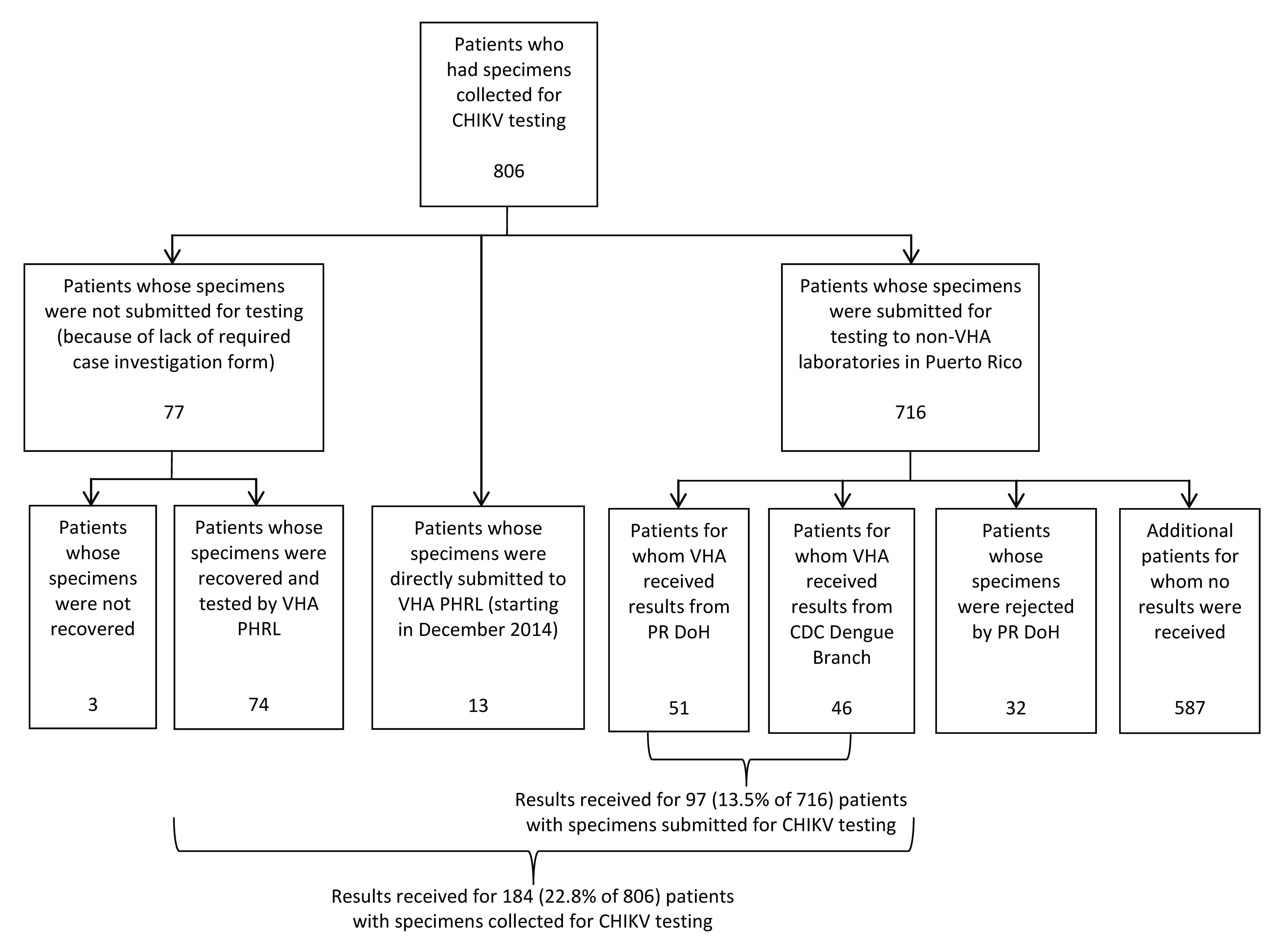

Supplement: S1 Fig — Of 806 patients who had specimens collected for chikungunya virus testing, 184 (22.8%) were tested. Of 716 patients who had specimens submitted to non-Veterans Health Administration laboratories in Puerto Rico for chikungunya virus testing, results were received for 97 (13.5%). Reasons provided for rejected specimens (n = 32) included incomplete information (symptom onset date or date of birth not specified), 29; no case investigation form, 1; discrepancy between specimen and form, 1; and unknown, 1. CHIKV, chikungunya virus; VHA, Veterans Health Administration; PHRL Public Health Reference Laboratory; and PR DoH, Puerto Rico Department of Health. (TIF) [file pntd.0004630.s001.tif]
